# Physician Burnout in General Hospitals Turned into Coronavirus Disease 2019 Priority Hospitals in Japan

**DOI:** 10.31662/jmaj.2021-0097

**Published:** 2021-12-15

**Authors:** Shiho Kodera, Yurika Kimura, Yutaka Tokairin, Hideaki Iseki, Makoto Kubo, Takayoshi Shimohata

**Affiliations:** 1 Department of Anesthesiology, Tokyo Metropolitan Health and Hospitals Corporation Ebara Hospital, Tokyo, Japan; 2 Department of Otolaryngology, Tokyo Metropolitan Health and Hospitals Corporation Ebara Hospital, Tokyo, Japan; 3 Department of Surgery, Tokyo Metropolitan Health and Hospitals Corporation Toshima Hospital, Tokyo, Japan; 4 Department of Surgery, Tokyo Metropolitan Hiroo Hospital, Tokyo, Japan; 5 Faculty of Policy Studies, Doshisha University, Kyoto, Japan; 6 Department of Neurology, Gifu University Graduate School of Medicine, Gifu, Japan

**Keywords:** COVID-19, physician, burnout, Japanese Burnout Scale, pandemic, contributing factor

## Introduction

The COVID-19 pandemic has added to already high levels of stress among medical professionals ^(1), (2), (3), (4)^. In January 2021, the Tokyo area was under pressure to provide more hospital beds for COVID-19 patients. Therefore, the metropolitan government decided to turn three public hospitals into COVID-19 treatment centers. These three hospitals subsequently suspended operation of some other treatment sections.

The unprecedented nature of the situation might have caused job stress and, consequently, burnout ^(5)^. As physician burnout is linked to their quality of life and reduced quality of care ^(6), (7)^, this study aimed to analyze the factors contributing to burnout among physicians whose environments changed rapidly due to the COVID-19 pandemic.

## Methods

We conducted an online, cross-sectional survey among all full-time doctors between February 15 and March 5, 2021, at the three regional hospitals that were redesignated as COVID-19 treatment centers, Tokyo Metropolitan Hiroo Hospital, Ebara Hospital, and Toshima Hospital of the Tokyo Metropolitan Health and Hospitals Corporation. We used Google Forms as the survey platform. The third wave of COVID-19 had begun to subside, although Tokyo had the highest numbers of infections in Japan, and was under a state of emergency. These three hospitals are regional general hospitals in Tokyo and are designated training hospitals. The institutional review boards of the three hospitals approved this study (No. 0283, Rin-rin-jin 2-47, Jin-73). All participants provided informed consent.

The primary study outcome, burnout, was measured using the Japanese Burnout Scale (JBS), a 17-item questionnaire with three subscales: emotional exhaustion (EE), depersonalization (DP), and lack of personal accomplishment (PA). Responses are coded on a scale from 1 (strongly disagree) to 5 (strongly agree). Higher scores indicate increased burnout ^(7), (8)^. No studies have validated the set cut-off to indicate burnout definitively ^(7)^.

Quantitative data were analyzed using BellCurve for Excel (version 3.21; Social Survey Research Information Co., Ltd.). Statistical significance was *p* < 0.05, and all tests were two-tailed. We used a one-way analysis of variance to test for differences across multiple groups. Multiple regression analysis was performed using IBM SPSS Statistics 27.

## Results

Of the 313 full-time physicians at the three COVID-19 priority hospitals invited to participate in this survey, 161 (51.4%) responded. The subjects included a higher population of males (n = 104, 64.6%). The largest percentage of subjects were in their 30s (n = 52, 32.3%), followed by the 40s (n = 46, 28.6%). Given the variety of medical departments, we divided the participants into four categories: Internal Medicine (internal medicine and pediatrics), Surgery (general surgery, cardiac surgery, neurosurgery, orthopedic surgery, obstetrics and gynecology, urology, anesthesiology, and emergency department), Outpatient and Laboratory (dermatology, otolaryngology, ophthalmology, plastic surgery, rehabilitation, radiology, pathology, and others), and residents. Working hours per week were divided into three categories to determine workload: within 40 hours/week (legal working hours), within 50 hours/week (legal overtime hours), and 51 hours or more/week (non-legal working hours) (Table 1).

**Table 1.** Characteristics of Physicians at COVID-19 Priority Hospitals in Tokyo and Their Japanese Burnout Scale Scores (mean ± SD).

	No.	(%)	Emotional exhaustion	Depersonalization	Lack of PA
Total	161		3.02 ± 1.03	2.55 ± 0.96	3.55 ± 0.91
Men	104	(64.6)	2.90 ± 1.02	2.48 ± 0.99	3.52 ± 0.94
Women	57	(35.4)	3.24 ± 1.01	2.66 ± 0.91	3.59 ± 0.87

Age (years) <30	27	(16.8)	3.00 ± 1.09	2.43 ± 0.89	3.56 ± 0.82
30-39	52	(32.3)	3.17 ± 1.05	2.74 ± 0.89	3.72 ± 0.83
40-49	46	(28.6)	2.96 ± 0.97	2.53 ± 0.94	3.54 ± 1.01
50-59	26	(16.1)	3.12 ± 1.03	2.54 ± 1.21	3.41 ± 0.90
≥60	10	(6.2)	2.36 ± 0.89	1.93 ± 0.74	3.02 ± 0.98

Internal medicine	56	(34.8)	3.15 ± 1.12	2.59 ± 1.04	3.58 ± 0.92
Surgery	67	(41.6)	3.08 ± 0.94	2.75 ± 0.95	3.68 ± 0.92
Outpatient and laboratory services	30	(18.6)	2.57 ± 0.98	2.07 ± 0.75	3.21 ± 0.91
Residents	8	(5.0)	3.33 ± 1.00	2.33 ± 0.62	3.42 ± 0.45
					
Work hours <40	39	(24.2)	2.94 ± 1.07	2.62 ± 0.96	3.91 ± 0.86
41-50	53	(32.9)	2.84 ± 1.01	2.47 ± 0.97	3.57 ± 0.81
>80	69	(42.8)	3.21 ± 1.00	2.57 ± 0.97	3.32 ± 0.95
					
Meaningful work
Strongly disagree	39	(24.2)	3.85 ± 0.74	3.53 ± 0.82	4.43 ± 0.69
Disagree	42	(26.1)	3.08 ± 0.94	2.69 ± 0.60	3.77 ± 0.53
Neutral	41	(25.5)	2.76 ± 1.00	2.25 ± 0.71	3.45 ± 0.62
Agree	25	(15.5)	2.42 ± 0.90	1.82 ± 0.72	2.69 ± 0.70
Strongly agree	14	(8.7)	2.40 ± 0.81	1.54 ± 0.61	2.27 ± 0.40

Abbreviations: SD, standard deviation; PA, personal accomplishment

**Figure 1.**
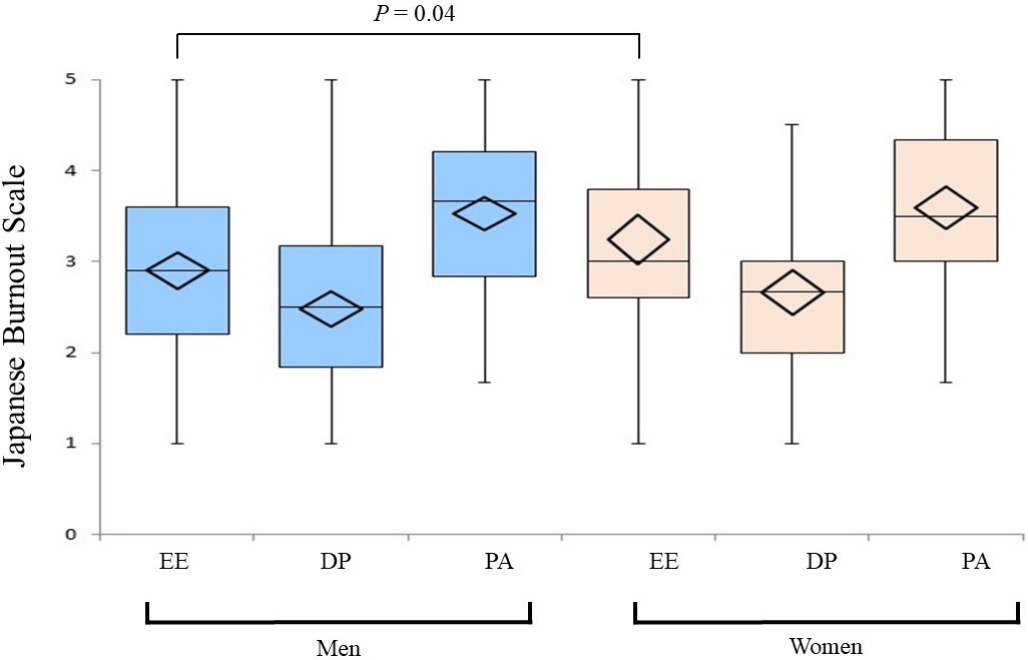
Sex differences in average scores on the Japanese Burnout Scale by subscale. The figure shows a box plot of the scores. The horizontal line in the box represents the median, and the diamond indicates the mean and standard deviation. There were significant differences between men (blue) and women (pink) in the emotional exhaustion subscale. Abbreviations: EE, emotional exhaustion; DP, depersonalization; PA, lack of personal accomplishment.

**Figure 2.**
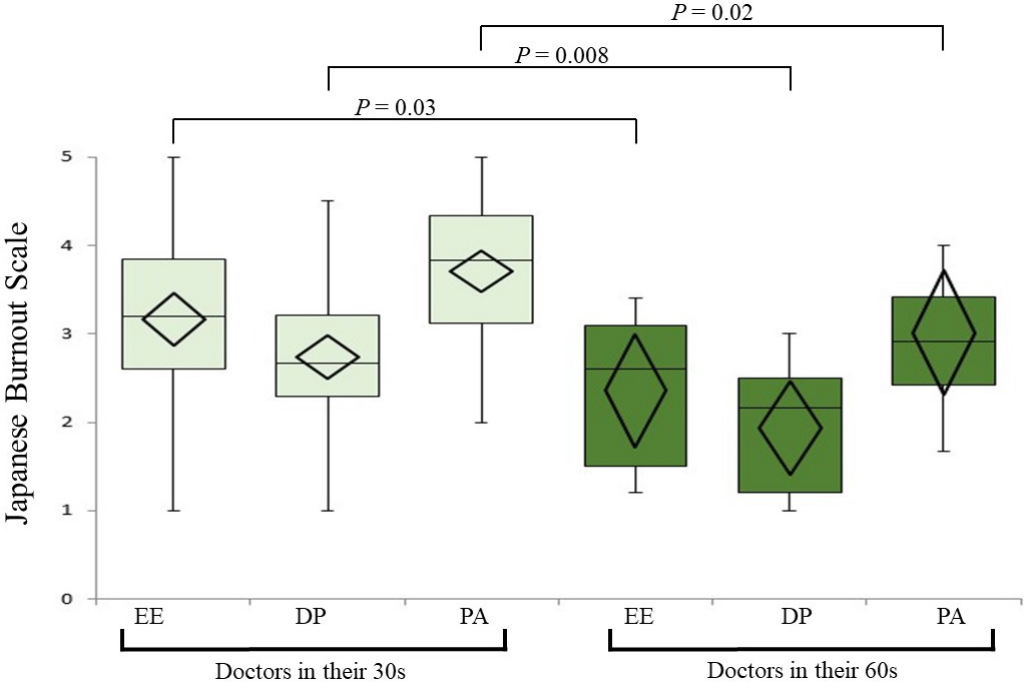
Relationship between age group and Japanese Burnout Scale (JBS) scores The figure shows a box plot of the JBS subscale scores of physicians in their 30s and 60s. The horizontal line in the box shows the median, and the diamond indicates the mean and standard deviation. Significant differences in subscale scores of the JBS appeared between doctors in their 30s (light green) and 60s (dark green). Abbreviations: EE, emotional exhaustion; DP, depersonalization; PA, lack of personal accomplishment.

**Figure 3.**
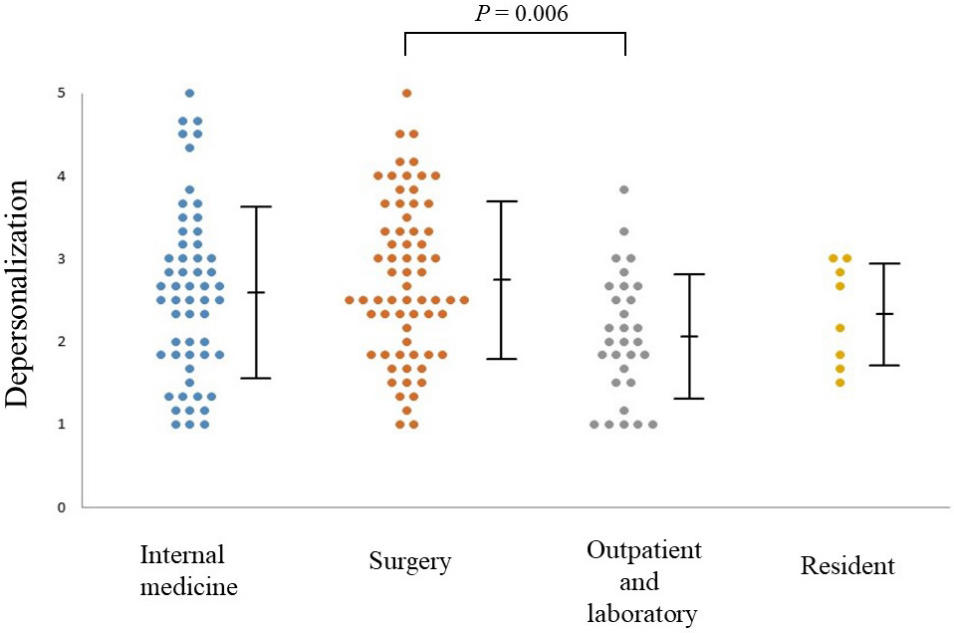
Dot plot diagram showing the correlation between departments and depersonalization scores on the Japanese Burnout Scale The sidebars show the mean and standard deviation. There were no differences in depersonalization scores between the internal medicine (blue) and surgery departments (red). However, there was a significant difference between the surgery and Outpatient and Laboratory Departments (gray).

The mean (± standard deviation) scores of the subject on the three JBS subscales were EE: 3.02 ± 1.03, DP: 2.55 ± 0.96, and PA: 3.55 ± 0.91. Figure 1 shows sex differences in the JBS subscale scores. Females tended to report higher EE than their male counterparts (3.24 vs. 2.90; *p* = 0.04). We found significant differences in all three JBS subscale scores between the groups in their 30s and those in their 60s, with physicians in their 30s expressing higher scores and hence, greater burnout (Figure 2). Figure 3 presents a dot plot diagram of the results by department. Outpatient and Laboratory Departments showed the lowest burnout rate. In terms of subscale scores, DP among surgery departments was higher than in Outpatient and Laboratory Departments (2.75 vs. 2.07; *p* = 0.006).

Table 2 shows multiple regression analysis using the stepwise method (input criterion: *p* <= 0.05, removal criterion: *p* >= 0.10) with the subscales of the JBS as objective variables and the items that showed significant associations. Four factors were associated with EE: meaningful work, work hours, sex, and change in workload after COVID-19. Work meaningfulness and work hours were related to DP, and the only factor associated with PA was work meaningfulness.

**Table 2.** Multiple Regression Analysis of the Japanese Burnout Scale’s Three Subscales.

**Emotional exhaustion**	
	Standardized partial regression coefficients (β)
Meaningful work	−0.567
dummy _ Work hours > 51 h	0.189
dummy _ Woman	0.177
Work change after COVID-19	0.164
	Adjusted R^2^ = 0.339
**Depersonalization**	
	Standardized partial regression coefficients (β)
Meaningful work	−0.697
dummy _ Work hours > 51 h	0.143
	Adjusted R^2^ = 0.470
**Lack of personal accomplishment**	
	Standardized partial regression coefficients (β)
Meaningful work	−0.780
	Adjusted R^2^ = 0.606

## Discussion

Freudenberger proposed the concept of burnout syndrome in a 1974 study of service workers, including nurses, teachers, and social workers ^(5), (7)^. Conventionally, physicians are considered to have a low probability of experiencing burnout because of their high level of professionalism and independence ^(7)^. However, with the COVID-19 pandemic, the social situation has changed rapidly, and the situation of physicians has also changed.

Baptista et al. surveyed 214 primary care physicians during the COVID-19 pandemic in Portugal and found that sex, years of experience, depression, and anxiety correlated strongly with burnout level ^(3)^. A survey of 1,961 Italian health care workers during the pandemic showed a tendency toward burnout among front-line residents, who were estimated to be highly burdened ^(4)^. We found some concordance with the literature on the influence of age on burnout ^(9), (10), (11), (12)^. Many of the young doctors in our sample were concerned that they would not be able to develop their careers owing to the pandemic cutting into their practice in their specialty. Although there have been many studies on sex differences in burnout rates, there is no definitive conclusion ^(9), (10)^. We speculated that female physicians might experience more EE than males because life events, such as pregnancy and childbirth, already cause them to have a shorter period for career development ^(9), (10), (13)^.

In a survey conducted by Shimohata et al. on all members of the Japanese Society of Neurology in 2019, among the 1,261 physicians who responded, mean scores on the JBS subscales were 2.86 points for EE, 2.21 points for DP, and 3.17 points for PA ^(14)^. Although all subscale scores in the present study were higher than neurologists in the previous study, the differences in DP and PA were particularly marked. In our study, EE, DP, and PA showed high standard partial regression coefficients for work meaningfulness. For physicians, saving patients by practicing their medical specialty leads to a feeling of accomplishment. Therefore, it is stressful for them to have only limited practice in their specialty ^(5), (7)^. As previously suggested, an increase in burnout in physicians could be related to the lack of meaningful work in a situation where their expertise in their specialty cannot be utilized ^(5), (15), (16)^.

The COVID-19 pandemic is a global disaster that has left administrators no choice but to accommodate the changes in the functionality of their hospital. Meanwhile, the stress on the employed staff is immeasurable ^(1), (2), (3), (4), (17)^. Our survey results on burnout identified women and younger doctors as the groups that felt more stressed. Our results also showed that loss of meaningfulness in their work can quickly lead to burnout among physicians, and the work environment of COVID-19 priority hospitals is a hotbed for this risk ^(5), (15)^. Burnout not only reduces the quality of medical care ^(6)^, but can also result in the loss of medical staff due to increased staff turnover ^(5)^. Our survey is limited by the fact that it was a one-time survey. It is hoped that further research will reduce burnout risk among physicians working at COVID-19 priority hospitals.

## Article Information

### Conflicts of Interest

None

### Author Contributions

YK created the research plan. SK drafted the manuscript. SK, YK and MK engaged in making the questionnaire. SK and YK collected data. All authors have revised and approved the final version.

### Approval by Institutional Review Board (IRB)

Tokyo Metropolitan Health and Hospitals Corporation Ebara Hospital approved this study (No.0283), Tokyo Metropolitan Health and Hospitals Corporation Toshima Hospital (Rin-rin-jin 2-47), and Tokyo Metropolitan Hiroo Hospital (Jin-73).
